# Anti-Inflammatory and Anti-Oxidant Effects of *Chlorella vulgaris* in an Experimental Acute Peritonitis Model

**DOI:** 10.3390/biomedicines14040878

**Published:** 2026-04-12

**Authors:** Yildirimcan Demirtas, Husnu Cagri Genc, Mustafa Ozkaraca, Mahmut Sahin, Alper Serhat Kumru, Atilla Kurt

**Affiliations:** 1 Department of General Surgery, Faculty of Medicine, Sivas Cumhuriyet University, 58140 Sivas, Türkiye; ydemirtas@cumhuriyet.edu.tr (Y.D.); atillakurt@cumhuriyet.edu.tr (A.K.); 2 Faculty of Veterinary Medicine, Sivas Cumhuriyet University, 58140 Sivas, Türkiye; mustafaozkaraca@cumhuriyet.edu.tr (M.O.); mahmutsahin@cumhuriyet.edu.tr (M.S.); askumru@cumhuriyet.edu.tr (A.S.K.)

**Keywords:** *Chlorella vulgaris*, peritonitis, cytokines, oxidative stress, anti-bacterial, anti-inflammatory

## Abstract

**Objectives**: Acute peritonitis remains a critical condition with high mortality rates, further complicated by the rising antibiotic resistance. This study aimed to investigate the therapeutic potential of *Chlorella vulgaris* (CHL), both alone and in combination with standard antibiotic therapy (SFT), in a rat model of cecal ligation and puncture (CLP)-induced peritonitis. **Methods**: Seventy (70) male Wistar albino rats were divided into seven groups (*n* = 10): Control, Peritonitis Control, Low-dose CHL (CHL I) (150 mg/kg), High-dose CHL (CHL II) (300 mg/kg), the standard first-line therapy group (SFT) (Ceftriaxone + Metronidazole), SFT + CHL I, and SFT + CHL II. Following CLP-induced peritonitis, treatments were administered for 7 days. Peritoneal tissues were evaluated histopathologically and immunohistochemically for TNF-α, IL-1β, IL-10, and iNOS expression. Total Antioxidant Status (TAS) and Total Oxidant Stress (TOS) were measured to assess the oxidative stress. **Results**: Histopathological analysis showed that CLP-induced severe inflammatory damage was significantly reduced in all treatment groups, with the most prominent recovery observed in the SFT + CHL II group. CHL treatment led to a significant decrease in pro-inflammatory markers (TNF-α, IL-1β, iNOS) compared to the peritonitis control group (PC) (*p* < 0.05). Furthermore, CHL administration significantly improved the oxidative balance by increasing TAS and reducing TOS levels. **Conclusions**: *Chlorella vulgaris* exhibits significant anti-inflammatory, antioxidant, and immunomodulatory properties in experimental peritonitis. When used as an adjunct to standard antibiotic therapy, high-dose CHL provides synergistic effects that contribute to limiting tissue damage and controlling systemic inflammation. These findings suggest that CHL may be a promising supportive agent in the clinical management of acute peritonitis.

## 1. Introduction

Acute peritonitis is an inflammatory condition of the peritoneum, most commonly caused by polymicrobial intra-abdominal infection. The resulting contamination of the peritoneal cavity triggers a complex pathophysiological cascade characterized by activation of the innate immune response, release of pro-inflammatory cytokines, endothelial dysfunction, and systemic inflammatory response. If uncontrolled, this process may progress to immune dysregulation, sepsis, multiple organ failure, and mortality [1,2]. Conventional treatment strategies involve surgical source management and broad-spectrum antibiotic administration. Antibiotic treatment should be administered empirically and targeted based on culture results. Peritonitis is typically caused by polymicrobial flora, including aerobic Gram-negative and anaerobic bacteria. International treatment guidelines recommend combination therapy with a third-generation cephalosporin and metronidazole to achieve broad-spectrum antimicrobial coverage [3,4]. Fluid and electrolyte monitoring and appropriate nutritional support are the mainstays of treatment.

The growing problem of antimicrobial resistance highlights the need for novel anti-infective agents. Natural products, including microalgae-derived compounds such as *Chlorella vulgaris* (CHL), have attracted attention as potential sources of antimicrobial and immunomodulatory agents with therapeutic potential in inflammatory and infectious diseases [5,6,7,8]. Microalgae, which are widely distributed in nature, have attracted increasing attention due to their potential therapeutic properties. Among them, CHL is well recognized for its potent anti-inflammatory, anti-oxidant, and immunomodulatory effects, largely attributed to its rich content of chlorophyll, carotenoids, polyunsaturated fatty acids, vitamins, and essential minerals [5]. Additionally, it contains high levels of vitamins A (beta-carotene) and E (tocopherols), which function as antioxidants [5]. CHL not only provides these vital vitamins but also serves as a significant source of essential minerals, including iron, calcium, magnesium, and zinc, all of which contribute to its health-promoting properties. The high iron content, in particular, has made CHL a potential supplement for individuals with iron deficiencies [9]. The antioxidant potential of CHL is largely attributed to its diverse array of bioactive compounds, including chlorophyll, carotenoids (such as lutein and β-carotene), polysaccharides, and polyphenols. These compounds collectively contribute to neutralizing harmful reactive oxygen species (ROS) and reducing oxidative stress. CHL exhibits significant anti-inflammatory properties due to its bioactive compounds, particularly carotenoids and polysaccharides. These compounds help suppress inflammatory pathways by reducing the production of pro-inflammatory cytokines such as Tumor Necrosis Factor-alpha (TNF-α) and Interleukin-6 (IL-6) while also inhibiting the activity of inflammatory enzymes like Cyclooxygenase-2 (COX-2). CHL is known for its ability to enhance both innate and adaptive immune responses, largely through the activation of cytokine production. Its polysaccharides stimulate the release of key molecules, which are essential for activating immune cells such as natural killer cells and T cells [5]. The anti-inflammatory and antioxidant effects of CHL are thought to be mediated through modulation of the NF-κB and Nrf2 signaling pathways. Suppression of NF-κB activation reduces transcription of pro-inflammatory mediators such as TNF-α, IL-1β, and iNOS, whereas activation of the Nrf2 pathway enhances cellular antioxidant defense mechanisms, including the expression of HO-1 and superoxide dismutase [10]. Additionally, Fihri et al. demonstrated that CHL has significant antimicrobial activity against Gram-negative bacteria, such as *Escherichia coli* and *Pseudomonas aeruginosa*, and Gram-positive bacteria, such as *Staphylococcus aureus* and *Bacillus subtilis*, in vitro [11]. CHL has been reported to have beneficial effects in various local inflammatory disease models, including hepatic injury [12], colitis [13], and wound healing [14].

However, evidence regarding the role of CHL in acute peritonitis, a condition characterized by polymicrobial infection, systemic immune activation, and widespread peritoneal inflammation, remains limited, especially in vivo. In particular, when CHL is used as an adjunct to antibiotic therapy, its effects on immune regulation, oxidative balance, and histopathological inflammatory damage remain incompletely elucidated.

Although commonly recommended human supplementation doses of CHL are approximately 2–3 g/day, experimental toxicological studies have demonstrated substantially higher safety limits. Previous animal studies reported no hematological, biochemical, or histopathological toxicity following oral administration of *Chlorella vulgaris*, with a No Observed Adverse Effect Level (NOAEL) of 1000 mg/kg/day in mice [15,16]. Experimental dosing in animal models is typically selected to achieve measurable biological effects rather than to replicate nutritional supplementation doses. Therefore, the CHL doses used in the present study were chosen within the established non-toxic experimental range reported in the literature.

This study aimed to investigate the anti-inflammatory, antioxidant, and immunomodulatory effects of CHL in an experimental model of acute peritonitis. The goal was to investigate the dose-dependent variation (high dose, low dose) of this effect and its change when administered in addition to standard antibiotic treatment, which is used in clinical practice for acute peritonitis.

## 2. Materials and Methods

In this study, adult male Wistar albino rats weighing 250–300 g each were obtained from the University Experimental Research Center. The animals were housed at 21 ± 2 °C, with 50–60% relative humidity, a 12 h light/12 h dark cycle, and provided standard pellet feed and water ad libitum.

This study was conducted with the approval of the Sivas Cumhuriyet University Local Ethics Committee for Animal Experiments (approval date: 8 December 2023; approval number: 65202830-050.04.04-05). All experimental procedures were performed in accordance with the European Union Directive 2010/63/EU.

### 2.1. Experimental Model

An acute peritonitis model was created using the cecal ligation and puncture (CLP) method. Anesthesia was induced by intraperitoneal administration of ketamine (90 mg/kg) and xylazine (3 mg/kg). After aseptic laparotomy, the cecum was exposed and ligated at approximately 10% of the distal cecal length with a 3-0 silk suture, followed by three punctures at separate sites using a 22-gauge needle [17,18]. After the puncture, gentle pressure was applied to the cecum to extrude fecal material, ensuring patency of the puncture holes and reproducibility of the model. The cecum was then returned to the peritoneal cavity, and the abdomen was closed in two layers.

Animals (*n* = 70) were randomly divided into seven groups (*n* = 10 per group) using computer-generated randomization. The control group underwent laparotomy only, without ligation or puncture. The peritonitis control group (PC) underwent cecal ligation and puncture (CLP). CHL I underwent CLP and was orally administered 150 mg/kg (low dose) of CHL. CHL II was subjected to CLP and orally administered 300 mg/kg (high dose) CHL. The standard first-line therapy group (SFT) underwent CLP and intramuscular (IM) injection of the standard first-line therapy (SFT) protocol, which was administered as a combination of ceftriaxone (50 mg/kg) and metronidazole (20 mg/kg), as adapted from previously published experimental peritonitis protocols [19,20]. The ceftriaxone–metronidazole combination was chosen based on its frequent use as a first-line empirical regimen for mild-to-moderate intra-abdominal infections in clinical practice and the literature [4,17,18,21]. The SFT + CHL-I group underwent CLP and received a combination of ceftriaxone (50 mg/kg) and metronidazole (20 mg/kg) and oral doses of 150 mg/kg (low dose) CHL. The SFT + CHL-II group underwent CLP and received a combination of ceftriaxone (50 mg/kg) and metronidazole (20 mg/kg) and oral doses of 300 mg/kg (high dose) CHL (Table 1).

CHL powder (Broken cell-wall) was obtained by opening commercially available Solgar^®^ Chlorella capsules (Solgar Inc., Leonia, NJ, USA), and the contents were weighed to calculate the necessary low (150 mg/kg) and high (300 mg/kg) doses based on previous studies [22,23]. The structural analysis and components of the natural CHL material used in the study were analyzed using FTIR, HPLC, and UV-VIS methods. The content of the CHL used was discussed with support from previously reported analysis results in the literature, and the conclusions are presented in the “Supplementary Document.” Treatment was initiated 24 h after CLP to allow stabilization of intra-abdominal infection and systemic inflammatory response, thereby creating a mild-to-moderate peritonitis model (cecal ligation for surgical source management) rather than a severe peritonitis model, consistent with previous CLP-based experimental sepsis studies [4,17]. All treatments were initiated 24 h after CLP induction and continued once daily for 7 days. At the end of the 7-day treatment period, the animals were re-anesthetized, and laparotomy was performed to collect peritoneal tissues for histopathological and immunohistochemical analyses. Terminal blood samples were collected via inferior vena cava puncture under deep anesthesia for biochemical analyses, followed by euthanasia with an overdose of ketamine and xylazine.

Postoperative analgesia was not administered in order to avoid potential confounding effects of analgesic and anti-inflammatory agents on cytokine expression, oxidative stress parameters, and histopathological inflammatory responses evaluated in this study. Analgesic agents, particularly opioids and NSAIDs, are known to modulate immune responses and inflammatory cytokine expression, including TNF-α, IL-1β, and IL-6, which may confound experimental outcomes in sepsis and peritonitis models [24,25,26]. Animals were closely monitored throughout the study period for signs of distress, and humane endpoints were predefined in accordance with institutional animal ethics guidelines.

No mortality was observed in any of the experimental groups. This outcome is likely related to the CLP protocol used in our study, in which a small cecal ligation (approximately 10% of the distal cecum cecal ligation) was provided for surgical source management, and three punctures with a 22-gauge needle were performed to induce mild-to-moderate intra-abdominal infection rather than severe sepsis. This approach allowed the development of a reproducible inflammatory response while maintaining animal survival throughout the experimental period.

### 2.2. Histopathology

Necropsies were performed on the rats, and the excised peritoneum was fixed in 10% formalin solution. Tissues were processed through routine alcohol–xylene steps and embedded in paraffin blocks. Five-micrometer-thick sections were cut and stained with hematoxylin and eosin (H&E). Histopathological evaluations of the peritoneal samples were performed using light microscopy (Olympus, Tokyo, Japan), according to the modified scoring system described by Bozkurt et al. Parameters, including peritoneal thickness, neovascularization, hyperemia, mononuclear cell infiltration, and fibroblast activity, were scored as absent (−), mild (+), moderate (++), or severe (+++) [27]. The evaluations were performed by a pathologist who was blinded to the treatment groups.

### 2.3. Immunohistochemistry

Five-micrometer-thick sections were obtained from paraffin-embedded peritoneal tissue samples. The sections were deparaffinized in xylene, rehydrated through a graded alcohol series, and washed with phosphate-buffered saline (PBS). To inhibit endogenous peroxidase activity, the sections were incubated with 3% hydrogen peroxide (H_2_O_2_) for 10 min. Heat-induced epitope retrieval (HIER) was then performed using a retrieval solution (10 mM citrate buffer, pH 6.0) in a microwave oven for two cycles of 5 min each at 500 Watts. After blocking, the tissues were washed with phosphate-buffered saline (PBS) and incubated overnight at 4 °C with primary antibodies (1/200 dilution) against Interleukin-10 (IL-10), Tumor Necrosis Factor-alpha (TNF-α), Interleukin-1 beta (IL-1β), and inducible Nitric Oxide Synthase (iNOS). As the secondary detection system, the Large Volume Detection System: anti-Polyvalent HRP (Thermo Fisher, Waltham, MA, USA, Catalog no. TP-125-HL) was used according to the manufacturer’s instructions. Immunoreactivity was visualized using 3,3′-diaminobenzidine (DAB) as the chromogen. After counterstaining with Mayer’s hematoxylin, the sections were mounted using Entellan. Slides were examined under a light microscope, and immunopositivity was assessed semiquantitatively using a modified scoring system [28] as follows: (−) absent, (+) mild, (++) moderate, (+++) severe, and (++++) very severe. All assessments were performed by investigators blinded to treatment allocation.

### 2.4. Biochemical Analyses

In the peritonitis model, serum and tissue samples were analyzed to assess oxidative stress by measuring total oxidant status (TOS) [29] and total antioxidant status (TAS) [30]. Measurements were performed according to the manufacturer’s protocols using the Total Oxidant Status Assay Kit and Total Antioxidant Status Assay Kit (Rel Assay Diagnostics, Gaziantep, Turkey).

TOS determination is based on the spectrophotometric measurement of the colored complex formed by oxidant molecules with the ferroin–otosidin (ferric) complex. The oxidants in the samples oxidize ferrous ions to ferric ions, which then form a complex with xylenol orange under acidic conditions, resulting in a color change. The resulting color change was measured at 530 nm using a spectrophotometer. The results were expressed as micromolar H_2_O_2_ equivalents (μmol H_2_O_2_ equivalents/L).

TAS determination is based on the ability of antioxidants in the sample to neutralize the ABTS [2,2′-azinobis(3-ethylbenzothiazoline-6-sulfonic acid)] radical cation. The ABTS radical cation is generated by the reaction of metmyoglobin with hydrogen peroxide, producing a blue-green color. Antioxidants in the sample reduce this radical, thereby decreasing the color intensity. Absorbance changes were measured at 660 nm using a spectrophotometer (Shimadzu, Kyoto, Japan). The results are expressed as Trolox equivalents (mmol Trolox equivalents/L). Both analyses were conducted in triplicate for all samples, and the mean values were used for statistical analyses.

### 2.5. Statistical Analysis

Statistical analyses were performed using SPSS software, version 20.0 (IBM Corp., Armonk, NY, USA). Biochemical data were analyzed using parametric methods: one-way ANOVA followed by Tukey’s post hoc test (*p* < 0.001). Histopathological and immunohistochemical data were analyzed using nonparametric tests: the Kruskal–Wallis test to assess differences among groups and the Mann–Whitney U test for pairwise comparisons (*p* < 0.05).

## 3. Results

All rats completed the study protocol. Therefore, data from all animals were included in the analysis.

### 3.1. Histopathological Findings

**Figure 1:**
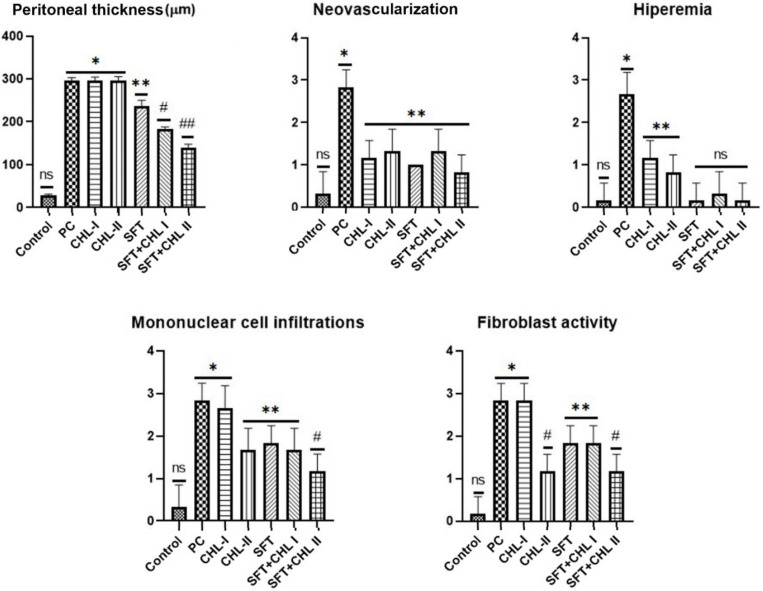
Statistical analysis of histopathological changes observed in the peritoneum. Overall group differences were highly significant for peritoneal thickness (*p* < 0.001, Kruskal–Wallis), while neovascularization, hyperaemia, mononuclear cell infiltration, and fibroblast activation showed significant variation among groups (*p* < 0.05). Post hoc pairwise differences (Mann–Whitney U) are indicated by *, #, and ##. “ns” denotes non-significant comparisons. (ns: not significant (*p* > 0.05), *: *p* < 0.05, Control group vs. PC group, **: *p* < 0.05, PC group vs. treatment groups, #: *p* < 0.001, PC group vs. treatment groups, ##: *p* < 0.001, PC group vs. SFT + CHL II group in peritoneal thickness).

**Figure 2:**
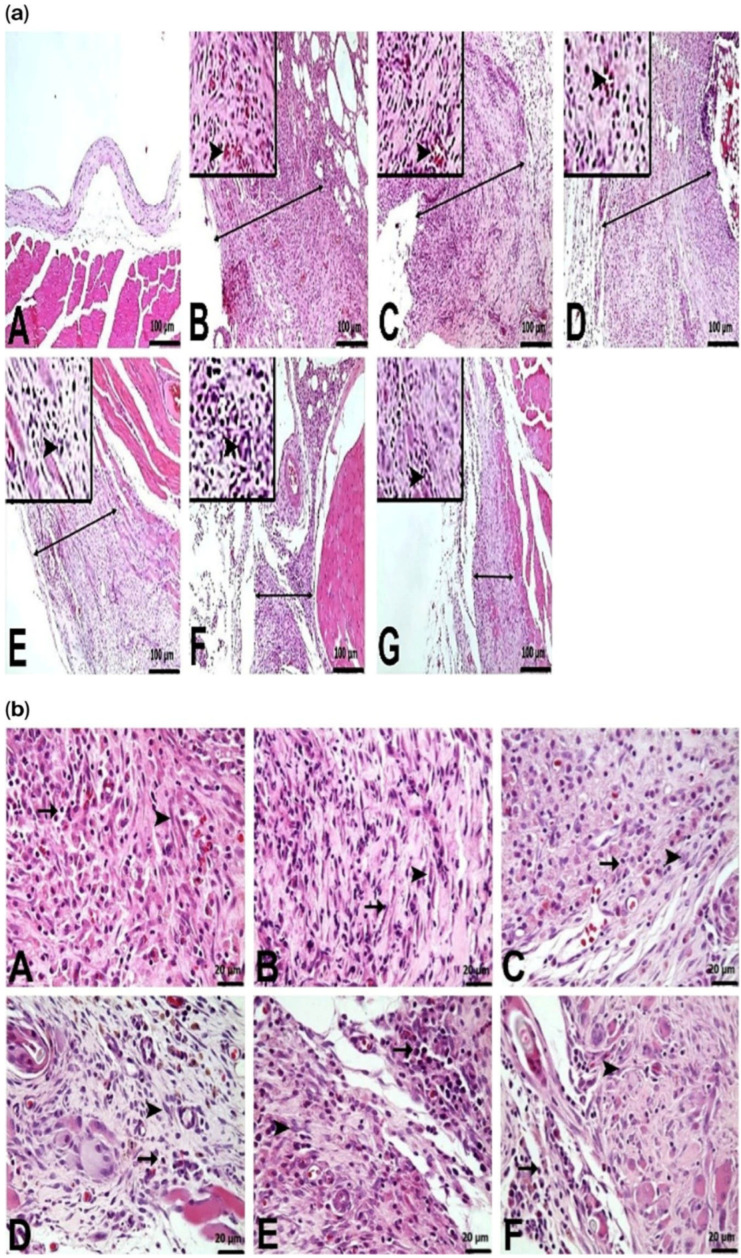
Histopathological appearance of the peritoneum. (200× magnification). (**a**) Peritoneal thickness, neovascularization, and hyperaemia. (**A**) Control group: Normal histological appearance. (**B**) PC group: Severe peritoneal thickness (↔) and severe neovascularization with hyperaemia (►). (**C**) CHL-I group and (**D**) CHL-II group. Severe peritoneal thickness (↔) and mild neovascularization with hyperaemia (►). (**E**) SFT group and (**F**) SFT + CHL-I group. Moderate/mild peritoneal thickness (↔) and mild neovascularization (►). (**G**) SFT + CHL-II group. Mild peritoneal thickness (↔) and mild neovascularization (►). Peritoneum, H&E. (**b**) Fibroblastic activity and mononuclear cell infiltration. (**A**) PC group and (**B**) CHL-I group Severe Fibroblastic activity (↔) and severe mononuclear cell infiltration (►). (**C**) CHL-II group, (**D**) SFT group and (**E**) SFT + CHL-I group. Moderate Fibroblastic activity (↔) and moerate mononuclear cell infiltration (►). (**F**) SFT + CHL-II group. Mild Fibroblastic activity (↔) and mild mononuclear cell infiltration (►) Peritoneum, H&E.

Statistically significant differences in histopathological findings were observed between the groups. The peritoneal samples from the control group exhibited a normal histological appearance. Peritoneal thickness increased in the PC (295.83 ± 7.03 µm), CHL-I (296.17 ± 8.33), and CHL-II (296.00 ± 9.51 µm) groups compared to the control group (28.67 ± 2.80 µm) (*p* < 0.05). There was a statistically significant difference between SFT and PC. Reduction in peritoneal thickness began in the SFT group (236.50 ± 13.58 µm). The thickness was moderate in the SFT + CHL-I group (184.00 ± 3.90 µm) and milder in the SFT + CHL-II group (139.67 ± 8.04 µm) (*p* < 0.001) (Figure 1 and Figure 2a).

Neovascularization and hyperemia were normal in the control group. Neovascularization and hyperemia were severe in the PC group, whereas they were mild in the CHL-I and CHL-II groups (*p* < 0.05). Hyperemia was observed to be mild in the SFT, SFT + CHL-I, and SFT + CHL-II groups. Mononuclear cell infiltration was severe in the PC and CHL-I groups, moderate in the CHL-II, SFT, and SFT + CHL-I groups, and mild in the SFT + CHL-II group (Figure 1 and Figure 2b). Mononuclear cell infiltration was statistically significantly reduced in the SFT + CHL-II group compared to other groups (*p* < 0.05). Fibroblastic activity in the PC and CHL-I groups was higher than that in the SFT and SFT + CHL-I groups. The lowest fibroblastic activity was observed in the CHL II and SFT + CHL II groups (Figure 1 and Figure 2b). Statistical values were explained in Figure 1 and Figure 2.

### 3.2. Immunohistochemical Findings

**Figure 3:**
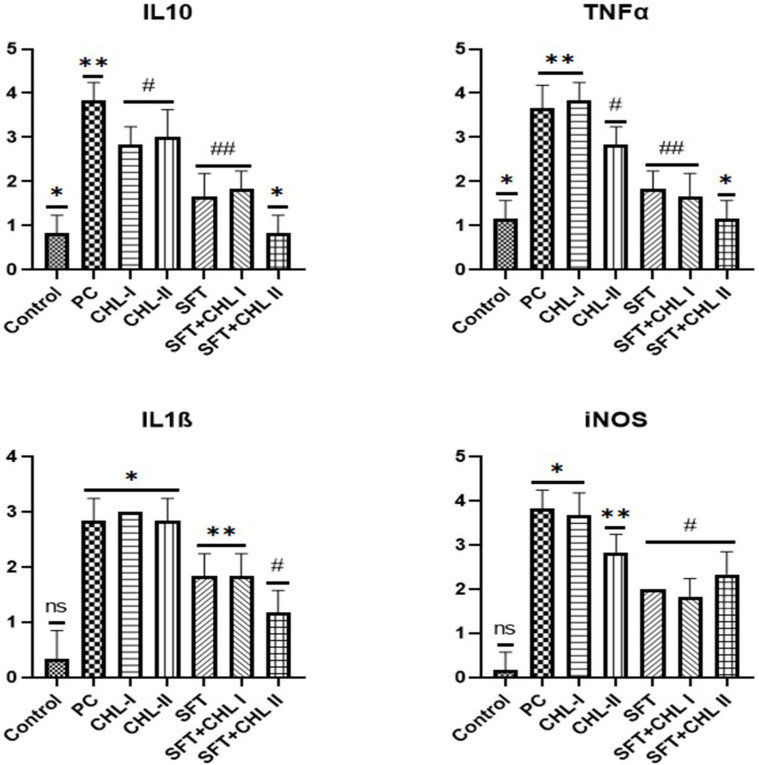
Semiquantitative immunohistochemical scoring of IL-10, TNF-α, IL-1β, and iNOS in peritoneal sections. Overall intergroup significance was tested by Kruskal–Wallis analysis (*p* < 0.05), followed by Mann–Whitney U post hoc testing. (ns: not significant (*p* > 0.05), *: *p* < 0.05, Control group vs. PC group, **: *p* < 0.05, PC group vs. treatment groups, #: *p* < 0.05, PC group vs. CHL I, CHL II, ##: *p* < 0.001, PC group vs. treatment groups).

**Figure 4:**
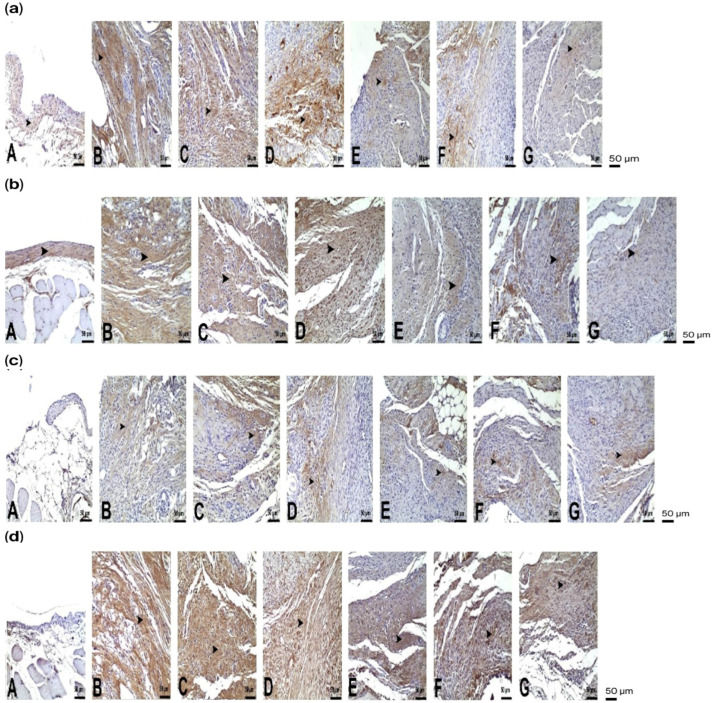
Representative immunohistochemical staining patterns of IL-10 (**a**), TNF-α (**b**), IL-1β (**c**), and iNOS (**d**) in peritoneal tissues across the studied groups: (**A**) Control (baseline staining), (**B**) PC (marked positivity/severe inflammation), (**C**) CHL-I (severe to moderate intensity), (**D**) CHL-II (severe to moderate intensity), (**E**) SFT (reduced staining compared to PC), (**F**) SFT + CHL I (partial attenuation), and (**G**) SFT + CHL-II (notable suppression with minimal residual staining). Arrows indicate positively stained inflammatory cells. Peritoneal sections, immunohistochemistry (IHC). (400× magnification).

Immunohistochemical staining for IL-10, TNF-α, IL-1β, and iNOS in peritoneal samples showed statistically significant differences among the groups. In peritoneal staining, immunopositivity was very severe in the PC group (*p* < 0.05). In the other treatment groups, CHL-I and CHL-II showed severe immunopositivity, SFT and SFT + CHL-I showed moderate immunopositivity, and SFT + CHL-II showed mild immunopositivity (Figure 3 and Figure 4).

### 3.3. Biochemical Findings

**Figure 5:**
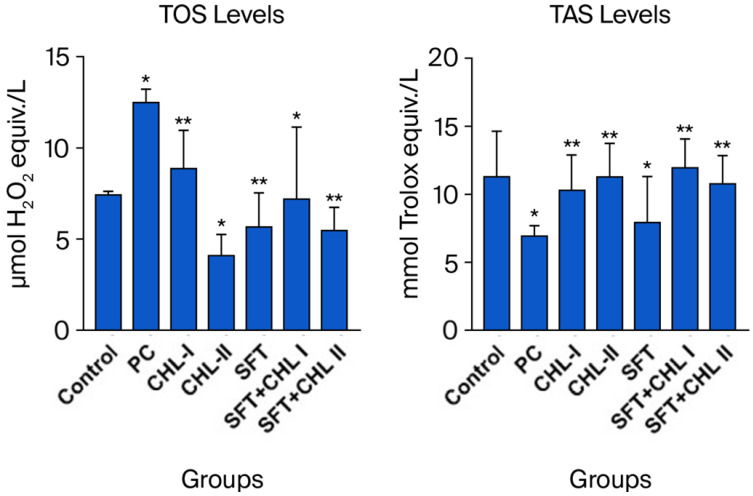
Comparison of total oxidative stress (TOS) and total antioxidant status (TAS) levels in experimental groups. *: *p* < 0.05, **: *p* < 0.001.

The lowest TOS levels were observed in the CHL-II and SFT + CHL II groups, which were significantly different from those in the other groups (* *p* < 0.05), especially compared to the PC group (Figure 5).

The highest TAS levels were statistically significantly observed in the CHL-I, CHL-II, SFT + CHL I, and SFT + CHL II groups compared to the PC group (* *p* < 0.05) (Figure 5).

## 4. Discussion

Acute peritonitis is associated with a high risk of systemic complications, morbidity, and mortality. This disease is primarily managed with prompt source control and broad-spectrum antibiotic therapy. However, antibiotic treatment cannot completely prevent inflammation and tissue damage [31]. Therefore, interest in adjunctive therapies with anti-bacterial, anti-inflammatory, immunomodulatory, and antioxidant properties has been increasing [32]. Fihi et al. [11] and Heo et al. [33] demonstrated the antibacterial activity of CHL in vitro. CHL has been shown to suppress proinflammatory cytokine release (TNF-α, IL-1β), modulate immune responses, and reduce oxidative stress, thereby accelerating tissue recovery. In this study, based on the literature, we evaluated the effects of CHL alone at different doses and in combination with standard antibiotic treatment using histopathological, immunohistochemical, and chemical analyses in an acute peritonitis model.

CHL accelerates the healing process by suppressing inflammatory cell migration [13]. Consistent with the literature, all inflammatory histopathological parameters (peritoneal thickness, neovascularization, hyperemia, mononuclear cell infiltration, and fibroblast activity) were significantly increased in the PC group compared to the control group. In the CHL-I and CHL-II groups, these parameters showed partial reductions because of the effect of different CHL doses; alone, treatment was observed to be limited. No significant differences were observed in peritoneal thickness, neovascularization, or hyperemia between CHL I and CHL II groups (*p* > 0.05). However, there were significant differences in mononuclear cell infiltration and fibroblast activity between CHL II and CHL I (*p* < 0.05). In contrast, the lowest values for all histopathological parameters were observed in the SFT + CHL II group (*p* < 0.001) (Figure 1). These results support the notion that high-dose CHL, when added to SFT, contributes to histopathological improvement in acute peritonitis.

In this study, IL-10, TNF-α, IL-1β, and iNOS cytokines were evaluated using immunohistochemistry. IL-10 plays a significant role in regulating the immune response by suppressing macrophage activation and reducing the production of proinflammatory cytokines. Previous studies have shown that CHL increases IL-10 expression, thereby reducing tissue damage in experimental models of colitis and sepsis [34]. Although studies have suggested that CHL increases IL-10 levels through its anti-inflammatory effects, our study observed a significant decrease in this cytokine (*p* < 0.05). This finding is consistent with data from Queiroz et al. and Hasegawa et al., which showed that *Chlorella* downregulates IL-10 to counteract its immunosuppressive effects [35,36]. The decrease in IL-10 levels in acute inflammatory processes, such as peritonitis, can be considered an indication that *Chlorella* shifts the pro-inflammatory/anti-inflammatory balance towards T helper 1 (Th1) cells to strengthen host defenses.

TNF-α, a central mediator of peritonitis pathogenesis associated with vascular permeability and tissue damage, was significantly elevated in the PC group compared with the control group (*p* < 0.05). CHL II administration partially reduced TNF-α levels, but the CHL II + SFT group showed the lowest TNF-α levels in our study (*p* < 0.05). Our findings are consistent with the literature [33,37].

IL-1β, a proinflammatory cytokine that initiates the inflammatory cascade and drives neutrophil chemotaxis [38], remained markedly elevated in the PC and CHL groups. In contrast, combined treatment with SFT + CHL-II significantly reduced IL-1β expression, demonstrating that the combination more effectively attenuates the inflammatory burden than either treatment alone.

Excessive nitric oxide derived from inducible Nitric Oxide Synthase (iNOS) is known to induce oxidative and nitrosative stress, leading to cellular injury [39]. iNOS levels were also significantly increased in the PC group (*p* < 0.05). In our study, the SFT + CHL I and SFT + CHL II groups markedly suppressed iNOS expression compared to other groups (*p* < 0.05). The absence of a statistically significant difference in iNOS levels between the antibiotic-treated groups may be explained by the anti-inflammatory effects of ceftriaxone and metronidazole, which are known to reduce infection-related inflammatory responses. In addition, the significantly lower iNOS expression observed in the high-dose CHL group compared with the peritonitis control group suggests that CHL alone may suppress iNOS expression, consistent with previous reports [40].

Oxidative stress refers to a shift in the balance of oxidative metabolism towards oxidation, resulting from an increase in oxidant levels and/or a decrease in antioxidant levels, cells, particularly in mitochondria, the nucleus, and the cell membrane. In CLP-induced peritonitis, polymicrobial infection activates innate immune responses, leading to excessive production of reactive oxygen species (ROS) and nitric oxide through iNOS activation. These mediators contribute to oxidative and nitrosative stress, endothelial dysfunction, and tissue injury. When antioxidant defense mechanisms are overwhelmed, oxidative damage becomes a central component of peritonitis-related organ injury. The antioxidant activity of *Chlorella vulgaris* is largely attributed to its chlorophyll and carotenoid content. In the analyses we presented in the Supplementary File regarding the content of CHL, the HPLC chromatogram revealed a distinct peak at approximately 8 min of retention time; this peak is likely associated with carotenoid derivatives such as violaxanthin found in Chlorella vulgaris. Violaxanthin is a well-known xanthophyll pigment in microalgae and is known to exhibit antioxidant and anti-inflammatory properties due to its ability to scavenge reactive oxygen species and regulate inflammatory pathways [41,42]. Therefore, the presence of such bioactive carotenoids and chlorophyll may contribute to the reduction in oxidative stress and inflammatory markers observed in the CHL-treated groups.

These compounds can directly scavenge reactive oxygen species, inhibit lipid peroxidation, and enhance endogenous antioxidant defense systems. Additionally, carotenoids and chlorophyll derivatives can indirectly reduce oxidative stress by suppressing inflammatory signaling pathways such as NF-κB and iNOS activation. Furthermore, chlorophyll and carotenoids activate superoxide dismutase (SOD) and catalase (CAT), which break down ROS [5]. Free radicals, which specifically affect DNA, can cause irreversible damage to the organism [43]. Oxidative damage occurs when oxidative stress increases and the antioxidant system is unable to compensate. In a review published by Chaudhary et al. [43], It was stated that synthetic antioxidants should be supplied from external sources to support the antioxidant defense mechanism to counteract oxidative stress. Owing to their therapeutic potential and natural origins, medicinal plants have been reported as the main source of natural antioxidant phytochemicals. Some non-enzymatic phytochemicals, such as flavonoids, polyphenols, and glutathione, as well as some vitamins, have been reported to have strong antioxidant activities in vivo and in vitro [5]. The CHL we used in our study is a natural medicinal plant that contains chlorophylls and carotenoids, as well as some vitamins and essential minerals. In our study, the CHL-II group showed reduced TOS and increased TAS. The combination of CHL and SFT improved these parameters.

The results of this study demonstrate that the combination of high-dose CHL and antibiotic therapy exerts significant anti-inflammatory effects and reduces oxidative stress in a model of experimental peritonitis. Our findings are consistent with the literature showing that CHL reduces pro-inflammatory cytokine levels, decreases oxidative stress, and accelerates histological healing in a peritonitis model [5,10,44]. In this study, we demonstrated that CHL exerted additive anti-inflammatory, antioxidant, and immunomodulatory effects when combined with standard antibiotic therapy in an experimental peritonitis model.

## 5. Conclusions

The study shows that, due to the anti-inflammatory, antioxidant, and immunomodulatory properties of CHL, it contributes to limiting tissue damage and controlling systemic inflammation. CHL treatment, particularly at high doses, has been associated with reductions in inflammatory markers and oxidative stress parameters, suggesting a dose-dependent response. These effects likely occur through its bioactive components, including chlorophyll, carotenoids, and polysaccharides, which are known to affect oxidative stress, inflammatory pathways, and immunomodulatory activity. In this context, integrating CHL-supported treatment protocols into clinical practice may provide significant benefits, especially in cases of peritonitis where antibiotic resistance is widespread or the immune response is weakened.

## Figures and Tables

**Table 1:** Experimental Groups and Applied Treatments.

Group Code	Groups	Treatment Content	Dose	Route
Control	Control (laparotomy only)	Normal saline	2 mL	Oral
PC	Peritonitis Control (CLP)	Normal saline	2 mL	Oral
CHL-I	*Chlorella vulgaris* low dose	*Chlorella vulgaris*	150 mg/kg	Oral
CHL-II	*Chlorella vulgaris* high dose	*Chlorella vulgaris*	300 mg/kg	Oral
SFT	Antibiotics only	Ceftriaxone + Metronidazole	50 mg/kg + 20 mg/kg	IM
SFT + CHL-I	Antibiotics + *Chlorella vulgaris* low dose	Ceftriaxone + Metronidazole + *Chlorella vulgaris*	50 mg/kg + 20 mg/kg + 150 mg/kg	IM + Oral
SFT + CHL-II	Antibiotic + *Chlorella vulgaris* high dose	Ceftriaxone + Metronidazole + *Chlorella vulgaris*	50 mg/kg + 20 mg/kg + 300 mg/kg	IM + Oral

CLP: Cecal ligation and puncture, CHL: *Chlorella vulgaris*, SFT: Standard first-line therapy, IM: Intramuscular.

## Data Availability

Data are available from the corresponding author upon request due to ethical restrictions.

## Supplementary Materials

The following supporting information can be downloaded at: https://www.mdpi.com/article/10.3390/biomedicines14040878/s1, Figure S1: FTIR spectrum of CHL; Figure S2: UV-VIS spectrum of CHL; Figure S3: HPLC result of CHL; References [45,46,47,48,49,50,51,52,53,54,55] are cited in the supplementary materials.

## Abbreviations

CHL	*Chlorella vulgaris*
PC	peritonitis Control group
CLP	cecal ligation and puncture
SFT	standard first-line therapy group
TOS	total oxidant stress
TAS	total antioxidant status

## References

[1] Sartelli M., Coccolini F., Kluger Y., Agastra E., Abu-Zidan F.M., Abbas A.E.S., Ansaloni L., Adesunkanmi A.K., Atanasov B., Augustin G. (2021). WSES/GAIS/SIS-E/WSIS/AAST global clinical pathways for patients with intra-abdominal infections. World J. Emerg. Surg..

[2] Mazuski J.E., Tessier J.M., May A.K., Sawyer R.G., Nadler E.P., Rosengart M.R., Chang P.K., O’NEill P.J., Mollen K.P., Huston J.M. (2017). The Surgical Infection Society revised guidelines on the management of intra-abdominal infection. Surg. Infect..

[3] Sartelli M., Chichom-Mefire A., Labricciosa F.M., Hardcastle T., Abu-Zidan F.M., Adesunkanmi A.K., Ansaloni L., Bala M., Balogh Z.J., Beltrán M.A. (2017). The management of intra-abdominal infections from a global perspective: 2017 WSES guidelines. World J. Emerg. Surg..

[4] Sartelli M., Palmieri M., Labricciosa F.M. (2025). Antibiotics for intra-abdominal infections: When, which, how and how long?. Antibiotics.

[5] Mendes A.R., Spínola M.P., Lordelo M., Prates J.A.M. (2024). Chemical compounds, bioactivities, and applications of *Chlorella vulgaris* in food, feed and medicine. Appl. Sci..

[6] Spolaore P., Joannis-Cassan C., Duran E., Isambert A. (2006). Commercial applications of microalgae. J. Biosci. Bioeng..

[7] Panahi Y., Darvishi B., Jowzi N., Beiraghdar F., Sahebkar A. (2016). *Chlorella vulgaris*: A multifunctional dietary supplement with diverse medicinal properties. Curr. Pharm. Des..

[8] Kotrbáček V., Doubek J., Doucha J. (2015). The chlorococcalean alga *Chlorella* in animal nutrition: A review. J. Appl. Phycol..

[9] Gouveia L., Batista A.P., Sousa I., Raymundo A., Bandarra N.M. (2018). Microalgae in novel food products. Microalgae as Source of Biochemicals and Functional Ingredients.

[10] Barghchi H., Dehnavi Z., Nattagh-Eshtivani E., Alwaily E.R., Almulla A.F., Kareem A.K., Barati M., Ranjbar G., Mohammadzadeh A., Rahimi P. (2023). The effects of *Chlorella vulgaris* on cardiovascular risk factors: A comprehensive review on putative molecular mechanisms. Biomed. Pharmacother..

[11] Fihri R.F., Ez-Zoubi A., Mbarkiou L., Amar A., Farah A., Bouchamma E.O. (2024). Antibacterial and antioxidant activities of *Chlorella vulgaris* and *Scenedesmus incrassatulus* using natural deep eutectic solvent under microwave-assisted ultrasound. Heliyon.

[12] Farage A.E., Abdel-Kareem M.A., Taha M., Abubakr S., Helal N.E., Hendawy M., Elgendy H.A., Elmetwally A.A.-M., Mahfouz H., Baokbah T.A.S. (2025). Protective effect of *Chlorella vulgaris* against experimental hepatic ischemia–reperfusion injury by downregulating oxidative stress, inflammation, and apoptosis. Anat. Cell Biol..

[13] Velankanni P., Go S.-H., Jin J.B., Park J.-S., Park S., Lee S.-B., Kwon H.-K., Pan C.-H., Cha K.H., Lee C.-G. (2023). *Chlorella vulgaris* modulates gut microbiota and induces regulatory T cells to alleviate colitis in mice. Nutrients.

[14] Mehdinezhad N., Aryaeian N., Vafa M., Saeedpour A., Ebrahimi A., Mobaderi T., Fahimi R., Hezaveh Z.S. (2021). Effect of spirulina and *Chlorella* alone and combined on the healing process of diabetic wounds: An experimental model of diabetic rats. J. Diabetes Metab. Disord..

[15] Yadav M., Sharma P., Kushwah H., Sandal N., Chauhan M.K. (2022). Assessment of the toxicological profile of *Chlorella vulgaris* powder by acute and subacute oral toxicity studies in mice. J. Appl. Phycol..

[16] Himuro S., Ueno S., Noguchi N., Uchikawa T., Watanabe K. (2014). Safety evaluation of mutagenicity and toxicity of *Chlorella vulgaris* CK-22 in rats. Fundam. Toxicol. Sci..

[17] Rittirsch D., Huber-Lang M.S., Flierl M.A., Ward P.A. (2009). Immunodesign of experimental sepsis by cecal ligation and puncture. Nat. Protoc..

[18] Sjaastad F.V., Jensen I.J., Berton R.R., Badovinac V.P., Griffith T.S. (2020). Inducing experimental polymicrobial sepsis by cecal ligation and puncture. Curr. Protoc. Immunol..

[19] Laudanski K., Lapko N., Zawadka M., Zhou B.X., Danet-Desnoyers G., Worthen G.S. (2017). The clinical and immunological performance of a 28-day survival model of cecal ligation and puncture in humanized mice. PLoS ONE.

[20] Vandewalle J., Steeland S., Van Ryckeghem S., Eggermont M., Van Wonterghem E., Vandenbroucke R.E., Libert C. (2019). Cecal ligation and puncture-induced sepsis in TNFR1-deficient mice. Front. Immunol..

[21] Bush L.M., Levison M.E. (2025). Peritonitis and intraperitoneal abscesses. Mandell, Douglas, and Bennett’s Principles and Practice of Infectious Diseases.

[22] Mohd Azamai E.S., Sulaiman S., Mohd Habib S.H., Looi M.L., Das S., Hamid N.A.A., Ngah W.Z.W., Yusof Y.A.M. (2009). *Chlorella vulgaris* triggers apoptosis in hepatocarcinogenesis-induced rats. J. Zhejiang Univ. Sci. B.

[23] Aboumosalem H., Mokhbatly A.A.A., Goda W., Ghazy E.W., Abou Elazab M.F., Abdelatty A., Elbialy Z.I., Assar D.H. (2025). *Chlorella vulgaris* attenuates acetic acid-induced colitis in rats via NF-κB and caspase-3 inhibition while activating IL-10 expression. Egypt. J. Vet. Sci..

[24] Sacerdote P. (2006). Opioids and the immune system. Palliat. Med..

[25] Ninković J., Roy S. (2013). Role of the mu-opioid receptor in opioid modulation of immune function. Amino Acids.

[26] Demirel G., Celik I.H., Canpolat F.E., Erdeve O., Oguz S.S., Dilmen U. (2012). The effects of ibuprofen on sepsis parameters in preterm neonates. Early Hum. Dev..

[27] Bozkurt D., Cetin P., Sipahi S., Hur E., Nar H., Ertilav M., Sezak M., Duman S. (2008). The effects of renin–angiotensin system inhibition on regression of encapsulating peritoneal sclerosis. Perit. Dial. Int..

[28] Yang M., Lin H.B., Gong S., Chen P.Y., Geng L.L., Zeng Y.M., Li D.Y. (2014). Effect of Astragalus polysaccharides on TNF-α, IL-1β, and NFATc4 expression in a rat model of colitis. Cytokine.

[29] Erel O. (2005). A new automated colorimetric method for measuring total oxidant status. Clin. Biochem..

[30] Erel O. (2004). A novel automated direct measurement method for total antioxidant capacity using a new generation ABTS radical cation. Clin. Biochem..

[31] Bonomo R.A., Chow A.W., Abrahamian F.M., Bessesen M., Dellinger E.P., Edwards M.S., Goldstein E., Hayden M.K., Humphries R., Kaye K.S. (2024). 2024 clinical practice guideline update by the Infectious Diseases Society of America on complicated intra-abdominal infections. Clin. Infect. Dis..

[32] Redondo-Calvo F.J., Montenegro O., Padilla-Valverde D., Villarejo P., Baladrón V., Bejarano-Ramírez N., Galán R., Gómez L.A., Villasanti N., Illescas S. (2021). Thiosulfinate-enriched *Allium sativum* extract as an adjunct to antibiotic treatment of sepsis. Appl. Sci..

[33] Heo Y., Kim M.Y., Cho J.Y. (2026). *Chlorella vulgaris* as an integrative and alternative medicine. Integr. Med. Res..

[34] Wu S., Liu H., Li S., Sun H., He X., Huang Y., Long H. (2021). Immunomodulatory mechanisms of *Chlorella* exopolysaccharides on macrophages. Mar. Drugs.

[35] Queiroz M.L., da Rocha M.C., Torello C.O., de Souza Queiroz J., Bincoletto C., Morgano M.A., Romano M.R., Paredes-Gamero E.J., Barbosa C.M., Calgarotto A.K. (2011). *Chlorella vulgaris* restores bone marrow cellularity and cytokine production. Food Chem. Toxicol..

[36] Hasegawa T., Kimura Y., Hiromatsu K., Kobayashi N., Yamada A., Makino M., Okuda M., Sano T., Nomoto K., Yoshikai Y. (1997). Effects of *Chlorella vulgaris* extract on cytokine expression. Immunopharmacology.

[37] Sibi G., Rabina S. (2016). Inhibition of pro-inflammatory mediators by *Chlorella vulgaris*. Pharmacogn. Res..

[38] Dinarello C.A. (2009). Immunological and inflammatory functions of the interleukin-1 family. Annu. Rev. Immunol..

[39] Förstermann U., Sessa W.C. (2012). Nitric oxide synthases: Regulation and function. Eur. Heart J..

[40] Martins T., Barros A.N., Rosa E., Antunes L. (2023). Chlorophyll-rich foods and health benefits. Molecules.

[41] Del Campo J.A., García-González M., Guerrero M.G. (2007). Outdoor cultivation of microalgae for carotenoid production. Appl. Microbiol. Biotechnol..

[42] Takaichi S. (2011). Carotenoids in algae: Distributions, biosyntheses and functions. Mar. Drugs.

[43] Chaudhary P., Janmeda P., Docea A.O., Yeskaliyeva B., Abdull Razis A.F., Modu B., Calina D., Sharifi-Rad J. (2023). Oxidative stress and antioxidants in disease. Front. Chem..

[44] Shiraishi C., Kato H., Ogura T., Iwamoto T. (2024). Broad-spectrum antibiotic-induced liver injury: A retrospective study. Sci. Rep..

[45] Hadjoudja S., Deluchat V., Baudu M. (2010). Cell surface characterisation of Microcystis aeruginosa and Chlorella vulgaris. J. Colloid Interface Sci..

[46] Ciempiel W., Czemierska M., Szymańska-Chargot M., Zdunek A., Wiącek D., Jarosz-Wilkołazka A., Krzemińska I. (2022). Soluble extracellular polymeric substances produced by Parachlorella kessleri and Chlorella vulgaris: Biochemical characterization and assessment of their cadmium and lead sorption abilities. Molecules.

[47] Polat S., Kılıç Ö.F. (2025). Pyrolysis of Chlorella vulgaris: Kinetic analysis, advanced characterization, and bio-oil optimization. J. Environ. Chem. Eng..

[48] El-Naggar N.E.A., Hussein M.H., Shaaban-Dessuuki S.A., Dalal S.R. (2020). Production, extraction and characterization of Chlorella vulgaris soluble polysaccharides and their applications in AgNPs biosynthesis and biostimulation of plant growth. Sci. Rep..

[49] Serratos I.N., Avila-Paredes H.J., Hernández-Reséndiz I., Santamaría A., Bustos-Terrones V., Sánchez P.R., Saucedo-Castañeda G., Shulz J.M.E., Arrieta A., Sosa R. (2021). Entrapment of chlorophyll from Chlorella vulgaris and Chlorella protothecoides into microporous silica synthesized by a solgel method. J. Phys. Commun..

[50] Paiva E.M., Hyttinen E., Dönsberg T., Barth D. (2025). Biological contaminants analysis in microalgae culture by UV–vis spectroscopy and machine learning. Spectrochim. Acta Part A Mol. Biomol. Spectrosc..

[51] Balan R., Suraishkumar G.K. (2014). Simultaneous increases in specific growth rate and specific lipid content of Chlorella vulgaris through UV-induced reactive species. Biotechnol. Prog..

[52] Pantami H.A., Ahamad Bustamam M.S., Lee S.Y., Ismail I.S., Mohd Faudzi S.M., Nakakuni M., Shaari K. (2020). Comprehensive GCMS and LC-MS/MS metabolite profiling of Chlorella vulgaris. Mar. Drugs.

[53] Stramarkou M., Papadaki S., Kyriakopoulou K., Krokida M. (2017). Effect of drying and extraction conditions on the recovery of bioactive compounds from Chlorella vulgaris. J. Appl. Phycol..

[54] Cha K.H., Kang S.W., Kim C.Y., Um B.H., Na Y.R., Pan C.H. (2010). Effect of pressurized liquids on extraction of antioxidants from Chlorella vulgaris. J. Agric. Food Chem..

[55] Inbaraj B.S., Chien J.T., Chen B.H. (2006). Improved high performance liquid chromatographic method for determination of carotenoids in the microalga Chlorella pyrenoidosa. J. Chromatogr. A.

