# Supplementary material for: Anti-Inflammatory and Anti-Oxidant Effects of Chlorella vulgaris in an Experimental Acute Peritonitis Model
Source: Biomedicines. 2026 Apr 12;14(4):878. doi: 10.3390/biomedicines14040878 (PMC13112965; doi:10.3390/biomedicines14040878)
Supplement: Supplementary file 1 [file biomedicines-14-00878-s001.zip › biomedicines-4226635-supplementary.pdf]

Structural analyses and content of CHL

1. FTIR analyses of CHL

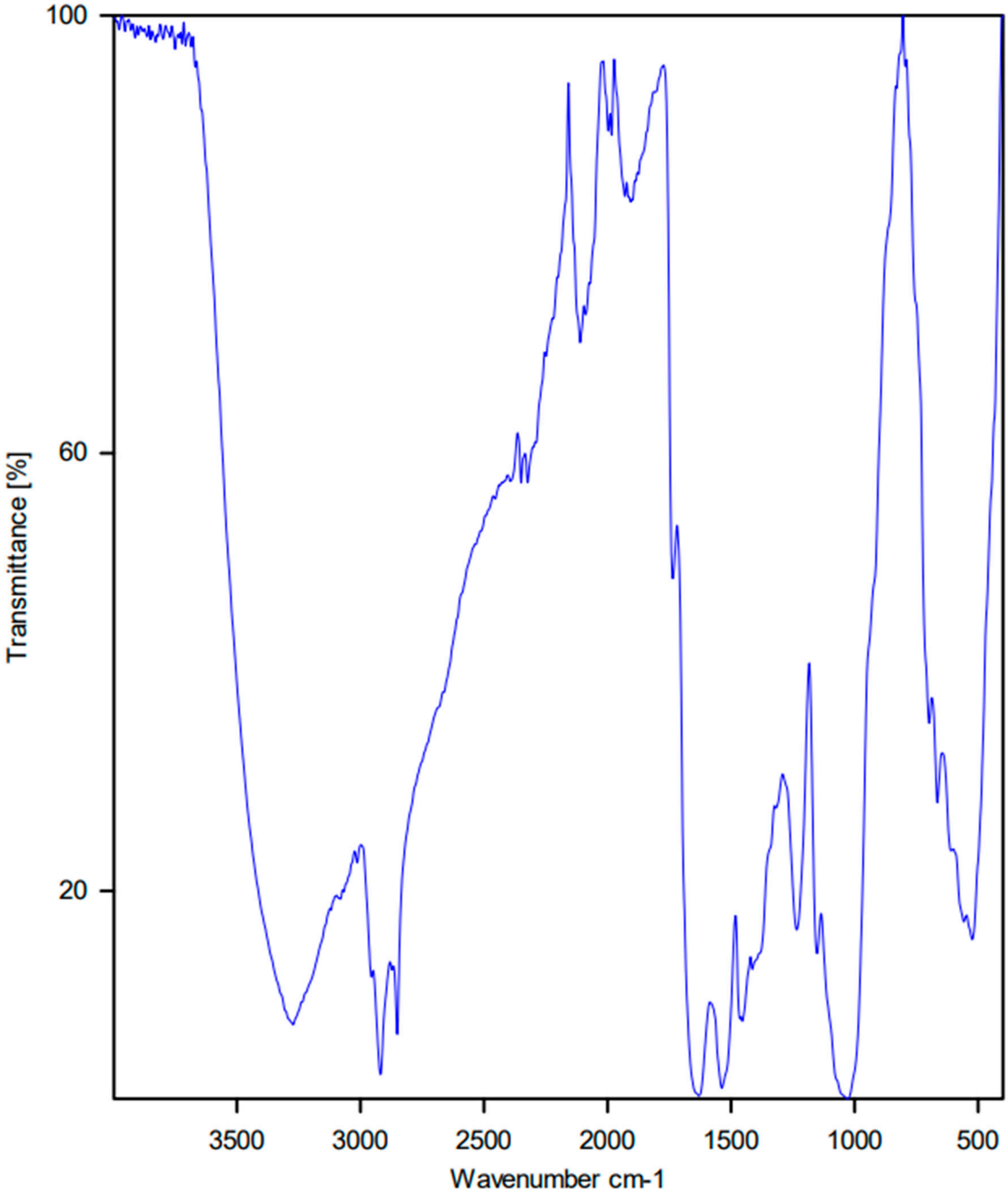

Figure S1. FTIR spectrum of CHL

The FTIR spectrum of the CHL sample used in this study is shown in figure 1. The wide peak at 3276 cm^{-1} corresponds to O-H and NH stretching vibrations and originates from polysaccharides and proteins present in the structure. The peaks at 2852-2920 cm^{-1} are characteristic peaks and correspond to symmetric-asymmetric C-H stretching vibrations in the

structure. The peak at 1735 cm^{-1} corresponds to C=O stretching vibrations and originates from ester groups in the lipid structure. In particular, the characteristic peaks of the ester bonds in the chlorophyll structure appeared in this region. The peaks at 1630 and 1538 cm^{-1} belong to amide I and amide II structures, respectively, and originate from the proteins in the CHL structure. The peak at 1151 cm^{-1} was evaluated as a characteristic peak of C-C bonds in the carotenoid structure related to stretching vibrations [1-4].

2. UV-VIS spectrum of CHL

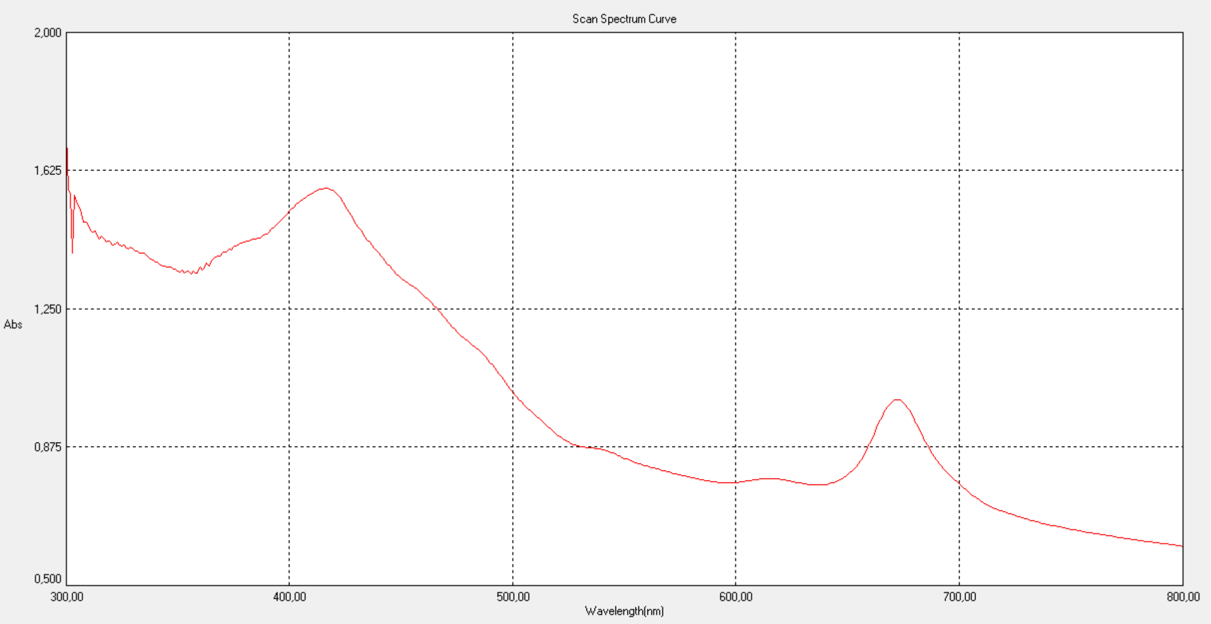

Figure S2. UV-VIS spectrum of CHL

The UV-Vis spectrum of the aqueous solution containing CHL is given in figure 2. In the UV-Vis spectrum in water, the broad peak at 410-430 nm, belonging to chlorophyll a, indicates the presence of chlorophyll, while the presence of chlorophyll b and carotenoids is considered the primary reason for the broadening of the peak. The band around 660 nm corresponds to the characteristic absorption peak of chlorophyll and has been evaluated as evidence that chlorophyll a is dominant in the CHL structure. The ridge between 450 and 490 nm is considered evidence of the presence of carotenoids [5-7].

3. HPLC results of CHL

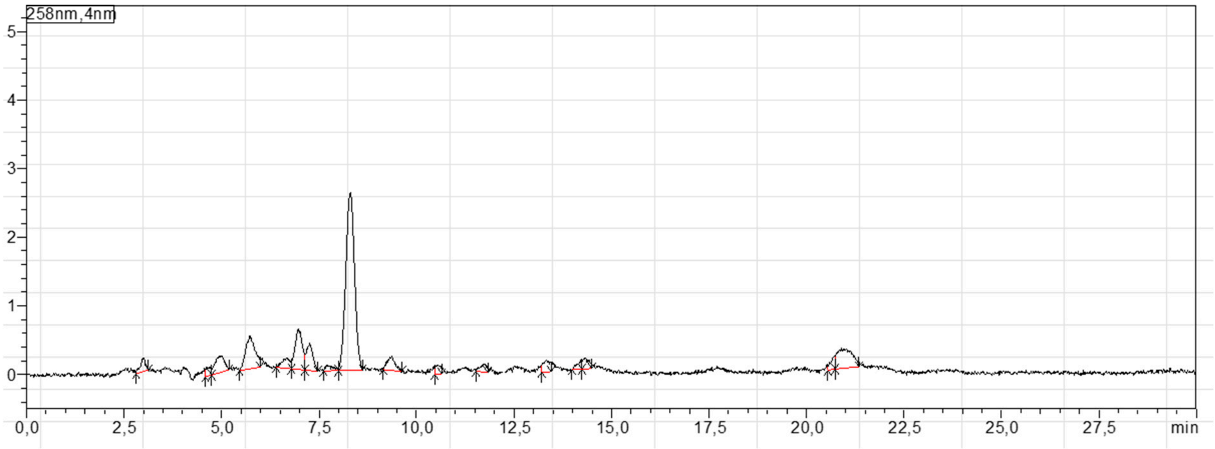

Figure S3. HPLC result of CHL

The HPLC analysis results of the CHL material solution in water are shown in Figure 3. The peaks observed between 2.5-7.5 minutes belong to numerous small-molecular-weight polar compounds, including pigments found in algae, such as luteoxanthin, loroxanthin, and lutein/siphonoxanthin. The intense peak that appears at the 8th minute has been identified as being due to violaxanthin, an active pigment found in the structure of CHL, a type of carotene. The molecules that appeared after 8 min had higher molecular weights and indicated the presence of phenolic compounds and carotenoid derivatives, β-zeacarotene, canthaxanthin, and β-carotene molecules. The molecule observed after 20 min was a chlorophyll residue, possibly Pheophytin a and b [9-11].

References

1. Hadjoudja, S., Deluchat, V., & Baudu, M. (2010). Cell surface characterisation of Microcystis aeruginosa and Chlorella vulgaris. *Journal of colloid and interface science*, *342*(2), 293-299.
2. Ciempiel, W., Czemierska, M., Szymańska-Chargot, M., Zdunek, A., Wiącek, D., Jarosz-Wilkołazka, A., & Krzemińska, I. (2022). Soluble extracellular polymeric substances produced by Parachlorella kessleri and Chlorella vulgaris: biochemical characterization and assessment of their cadmium and lead sorption abilities. *Molecules*, *27*(21), 7153.
3. Polat, S., & Kılıç, Ö. F. (2025). Pyrolysis of Chlorella vulgaris: Kinetic analysis, advanced characterization, and bio-oil optimization. *Journal of Environmental Chemical Engineering*, 120374.

4. El-Naggar, N. E. A., Hussein, M. H., Shaaban-Dessuuki, S. A., & Dalal, S. R. (2020). Production, extraction and characterization of Chlorella vulgaris soluble polysaccharides and their applications in AgNPs biosynthesis and biostimulation of plant growth. *Scientific Reports*, *10*(1), 3011.
5. Serratos, I. N., Avila-Paredes, H. J., Hernández-Reséndiz, I., Santamaría, A., Bustos-Terrones, V., Ruiz Sánchez, P., ... & Sosa, R. (2021). Entrapment of chlorophyll from Chlorella vulgaris and Chlorella protothecoides into microporous silica synthesized by a sol-gel method. *Journal of Physics Communications*, *5*(10), 105004.
6. Paiva, E. M., Hyttinen, E., Dönsberg, T., & Barth, D. (2025). Biological contaminants analysis in microalgae culture by UV–vis spectroscopy and machine learning. *Spectrochimica Acta Part A: Molecular and Biomolecular Spectroscopy*, *330*, 125690.
7. Balan, R., & Suraishkumar, G. K. (2014). Simultaneous increases in specific growth rate and specific lipid content of Chlorella vulgaris through UV-induced reactive species. *Biotechnology Progress*, *30*(2), 291-299.
8. Pantami, H. A., Ahamad Bustamam, M. S., Lee, S. Y., Ismail, I. S., Mohd Faudzi, S. M., Nakakuni, M., & Shaari, K. (2020). Comprehensive GCMS and LC-MS/MS metabolite profiling of Chlorella vulgaris. Marine drugs, 18(7), 367.
9. Stramarkou, M., Papadaki, S., Kyriakopoulou, K., & Krokida, M. (2017). Effect of drying and extraction conditions on the recovery of bioactive compounds from Chlorella vulgaris. *Journal of Applied Phycology*, *29*(6), 2947-2960.
10. Cha, K. H., Kang, S. W., Kim, C. Y., Um, B. H., Na, Y. R., & Pan, C. H. (2010). Effect of pressurized liquids on extraction of antioxidants from Chlorella vulgaris. *Journal of agricultural and food chemistry*, *58*(8), 4756-4761.
11. Inbaraj, B. S., Chien, J. T., & Chen, B. H. (2006). Improved high performance liquid chromatographic method for determination of carotenoids in the microalga Chlorella pyrenoidosa. *Journal of chromatography A*, *1102*(1-2), 193-199.
